# Analysis of the Screw Accuracy and Postoperative Efficacy of Screw Placement in Single Position and Bipedal Position in Robot‐Assisted Oblique Lumbar Interbody Fusion: Preliminary Results of Mazor X Stealth Usage

**DOI:** 10.1111/os.13972

**Published:** 2023-12-27

**Authors:** Wenhao Zhao, Yan Wang, Hao Zhang, Jianwei Guo, Jialuo Han, Antao Lin, Chuanli Zhou, Xuexiao Ma

**Affiliations:** ^1^ Department of Spine Surgery The Affiliated Hospital of Qingdao University Qingdao China

**Keywords:** Minimally invasive, Navigation, Pedicle screw accuracy, Robot, Robot assistance, Spine surgery

## Abstract

**Objective:**

Traditional manual OLIF combined with pedicle screw implantation has many problems of manual percutaneous screw implantation, such as high difficulty of screw placement, many fluoroscopies, long operation time, and many adjustments, resulting in greater trauma. The robot can perform various types of screw placement in the lateral recumbent position, which allows OLIF combined with posterior screw placement surgery to be completed in a single position. To compare the screw accuracy and initial postoperative results of oblique anterior lumbar fusion with robot‐assisted screw placement in the lateral position and screw placement in the prone position for the treatment of lumbar spondylolisthesis.

**Methods:**

From May to June 2022, 45 patients with single‐segment lumbar spondylolisthesis underwent Mazor X‐assisted oblique lumbar fusion in one position and Renaissance‐assisted surgery in two different positions, and screw accuracy was assessed on computed tomography scans according to a modified Gertzbein–Robbins classification. Patients were divided into a single position group and a bipedal position group (the lateral position for complete oblique lumbar fusion and then changed to the prone position for posterior screw placement), and the perioperative parameters, including operative time, number of fluoroscopies, and operative complications, were recorded separately. The results of the clinical indicators, such as the visual analog scale (VAS) for back and leg pain and the Oswestry Disability Index (ODI) score, were obtained.

**Results:**

There were no significant differences in the patients' demographic data between the two groups. The single position group had a shorter operative time and fewer fluoroscopies than the bipedal position group; the single position group had a higher percentage of screw accuracy at the A level than the bipedal position group, but there was no statistically significant difference between the two groups at the acceptable level (A + B) (*p* > 0.05). The single‐position group had better outcomes at the 1‐week postoperative follow‐up back pain VAS scores (*p* < 0.05). There was no statistically significant difference in the postoperative leg pain VAS scores or the ODI scores when compared to the control group.

**Conclusion:**

Robot‐assisted lateral position oblique lumbar interbody fusion with pedicle screw placement has the same accuracy as prone positioning. Single position surgery can significantly shorten the operation time and reduce the fluoroscopy. There was no significant difference in the long‐term efficacy between the two groups.

## Graphical abstract

The Mazor X robot assists in the placement of pedicle screws in a lateral position and displays the screw trajectory in real‐time on the display.

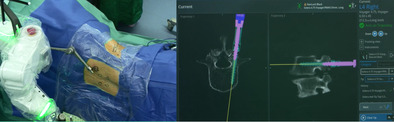

## Introduction

Oblique lumbar interbody fusion (hereinafter referred to as OLIF), compared with traditional posterior lateral approach lumbar fusion, allows access to the vertebral body through the physiological gap between the retroperitoneum and the psoas major muscle through a small surgical incision, thereby causing less damage to the muscles, ligament complex and bony structures in the posterior spine, which is conducive to ensuring the stability of the posterior spinal column. Moreover, this procedure does not interfere with the spinal canal and is thus less likely to cause a spinal cord or nerve injury.^1^ In contrast, for patients with osteoporosis, posterior screw bar fixation is required to prevent endplate collapse caused by OLIF after completion of oblique anterior treatment with a cage. Therefore, the intraoperative position of the patient needs to be changed from right lateral to prone, after which sterilization, sheeting, and positioning must be repeated, which are additional surgical steps that prolong the operative time.^2^

In recent years, with the technological development of image navigation and artificial intelligence, internal spinal fixators have been introduced. Surgeons have begun to experiment with various aids, such as electromagnetic navigation systems, robotics, and optical navigation, to help complete the surgery, reduce the difficulty of the procedure and improve the accuracy of screw placement.^3^, ^4^, ^5^ The use of robots during spine surgery can effectively reduce the surgeon's dependence on their ability to accurately recognize anatomical structures, as surgical experience is less considered.^6^, ^7^ The development of several navigation systems or robotic systems has made screw placement in the lateral position much less difficult.^8^, ^9^, ^10^ The Mazor X stealth robot (Medtronic, America, hereinafter referred to as the Mazor X robot), which was first introduced in China, can be used to perform oblique lumbar interbody fusion surgery and simultaneous posterior percutaneous screw placement in the lateral position, thereby shortening the operative time and reducing radiation exposure while improving the accuracy of screw placement.^11^

Since its inception, robot‐assisted spine surgery has been expected to improve the accuracy of screw placement, reduce radiation exposure, and reduce the difficulty of surgery.^7^, ^12^, ^13^ A multicenter study by Lee et al., including seven surgeons at four institutions, showed significant improvements in robotic screw accuracy, reliability, surgical efficiency, and radiation exposure from 2015 to 2019, with 90‐day complication rates remaining low.^14^ A systematic review and meta‐analysis of pooled data by Tovar et al. showed that robot‐assisted instrumentation was associated with a lower risk of inaccurate screw placement (*p* < 0.0001) regardless of the control arm approach (freehand, fluoroscopy guided, or navigation guided), fewer reoperations (*p* < 0.0001), fewer perioperative complications (*p* < 0.0001), lower EBL (*p* = 0.0005), shorter LOS (*p* < 0.0001), and longer intraoperative time (*p* = 0.0003).^15^ Compared with traditional dual‐position OLIF surgery for posterior screw reinforcement, unilateral robot‐assisted OLIF with screw placement has significant technical advantages, such as reduced fatigue, enhanced stability, and reduced radiation exposure.^16^

Here, we retrospectively collected the clinical data of patients who underwent robotic‐assisted OLIF surgery at our institution, with the aim of (I) comparing the advantages and disadvantages of Mazor X robot‐assisted unilateral OLIF with screw placement with Renaissance‐assisted bipedal position surgery, (II) summarizing our experience in performing this procedure to provide a basis for its promotion as a promising technique.

## Materials and Methods

### Study Methods and Patient Information

A retrospective case–control study was performed to compare the single position group with the bipedal position group. A total of 45 patients who were eligible for inclusion according to the screening criteria participated in this study between May and June 2022. The procedure was approved by the Affiliated Hospital of Qingdao University. The inclusion criteria were defined as follows: patients who were over 18 years of age^1^; patients were diagnosed with degenerative lumbar spondylolisthesis, under Meyerding Grade II, and conservative treatment for more than 3 months was ineffective^2^; patients had neurological symptoms (including low back pain, sciatica, and extremity symptoms)^3^; and patients underwent oblique lumbar interbody fusion with posterior pedicle screw placement.^4^ The exclusion criteria were defined as follows: severe spinal stenosis^1^; severe osteoporosis, morbid obesity (body mass index (BMI) >35 kg/m2)^2^; history of lumbar surgery, infection, tumor, or other serious diseases^3^; and preoperative segmental spontaneous fusion of the intervertebral space.^4^

We examined the patients' medical records and imaging information. The data were categorized as follows: general information, i.e., age, sex, and body mass index (BMI)^1^; surgical information, such as primary diagnosis, type of surgery (single or dual position), number of screws, type of screws, duration of surgery, EBL (estimated blood loss), and number of fluoroscopies^2^; and surgical efficacy information, i.e., postoperative assessment of screw placement accuracy and patients' VAS scores and ODI scores^3^. All patients provided informed consent preoperatively.

Our study was approved by the Ethics Committee of Affiliated Hospital of Qingdao University (Approval: QYFY WZLL 28119). All methods were carried out in accordance with relevant guidelines and regulations.

### Surgical Procedure

#### Single Position Group

##### Preoperative Planning

**FIGURE 1:**
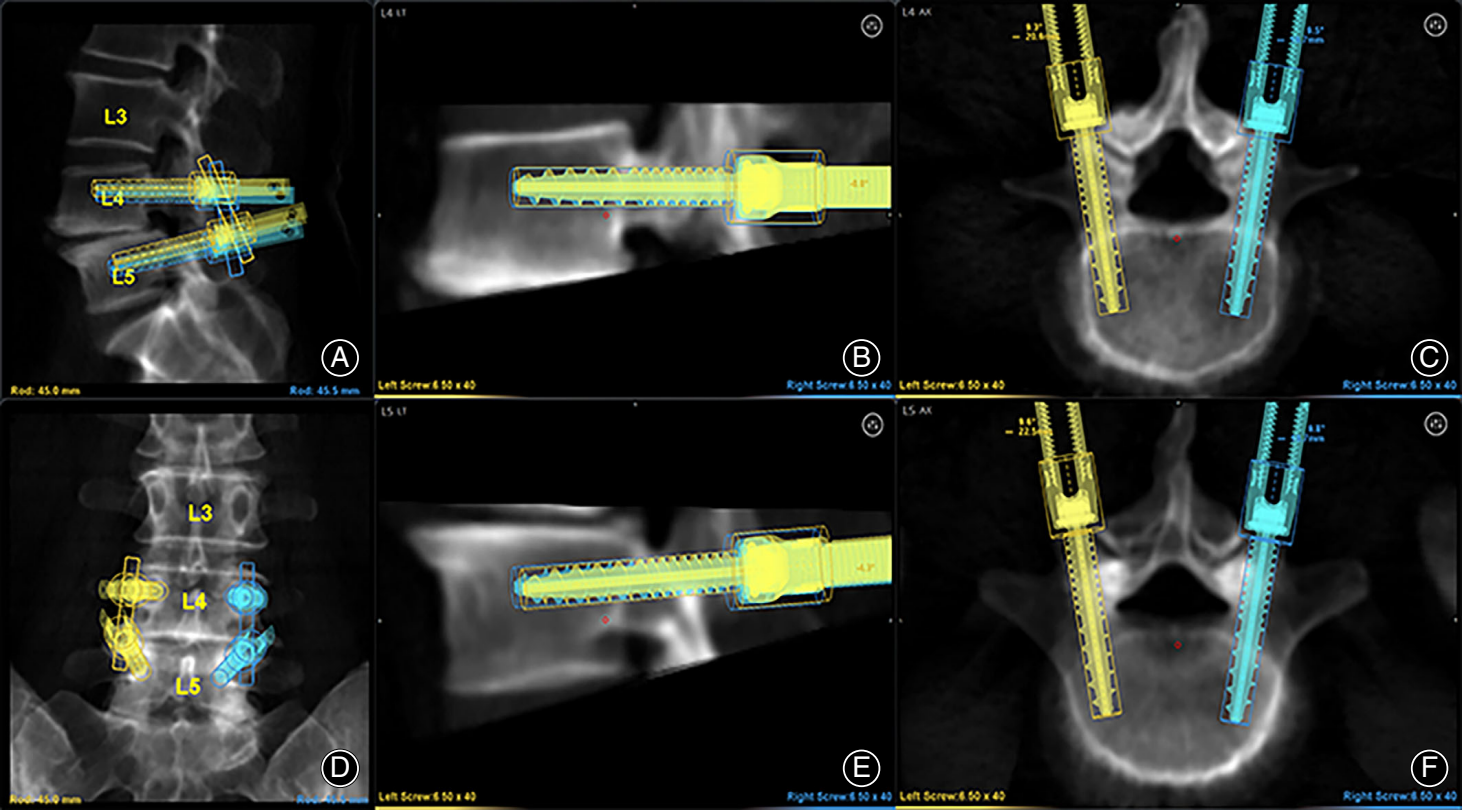
The interface of the Mazor X Stealth robot preoperative plan. (A) Lateral overview of the surgical segment. (B) L4 screw side position adjustment. (C) L4 screw coronal position adjustment. (D) Orthostatic overview of the surgical segment. (E) L5 screw side position adjustment. (F) L5 screw coronal position adjustment.

The robotic platform has two different approaches that the surgeon can choose from to perform the procedure: the intraoperative O‐ARM scan or the preoperative computed tomography (CT) scan. In the latter approach, the patient undergoes the same standard CT scan using the same model (<1 mm layer thickness, DICOM format), and the data are transmitted to the software of the Mazor X robot system, where the surgeon can select the type of screw (pedicle screw or cortical bone screw) in the sagittal, coronal, and transverse axes and make adjustments to the angle, length, and diameter of the virtual screw. The system automatically generates the length, bending angle, and shape of the virtual connecting rod according to the surgical plan (Figure 1). The surgical plan is transferred from the operating technician to the workstation prior to the start of the procedure.

##### Positioning

After induction of general anesthesia, the patient was placed in a right lateral position with the back of the trunk as close to the side of the surgical bed as possible to ensure that placement of the pedicle screw on the right side was not affected by the operating table. The patient was padded with a silicone pad at the L4‐S1 level to ensure that the surgical segment was in a horizontal position, and the patient's lower extremities were kept in a flexed hip and knee position to keep the psoas major relaxed. The patient was fixed with medical tape under the subacromial thorax and iliac crest to keep the position stable. Silicone pads and anti‐pressure sore patches were placed on each weight‐bearing area to prevent pressure sores.

##### Robot Installation, Registration

After completing the positioning mark, connect the robot to the bed rail adapter installed at the end of the operating table, and then disinfect and cover the surgical area. The robot's arms and tablets are covered with a customized sterile cover to ensure the cleanliness of the surgical area. The robot is connected to the patient in one of three ways, including a single posterior superior iliac spine screw, bilateral screws, spinal clips or multiple spinal head pins with connecting bridges that can be used for percutaneous surgery; The posterior superior iliac spine screw was used for fixation. After fixing, use the marker plate (fluorescent marker array) for fluorescent alignment in the orthogonal oblique position. After calibration, the patient remains relatively stationary to minimize jitter, so as not to affect accuracy. The next step is to match the navigation system. First, cover the surgical area with sterile surgical film, and then conduct three‐dimensional scanning of the surgical area with the built‐in optical camera on the robot arm, and then use the aligner to align tapping, binding and other tools. At this point, the preparations have been completed.

##### Power‐Assisted Screw Placement without Guide Wire

The screw to be placed was selected at the workstation, the robot arm was positioned, and a special 11‐gauge scalpel equipped with the robot was used for puncture. An incision measuring approximately 3–4 cm in length was made, and the deep fascia was sufficiently cut to prevent the screw from being placed in the fascia or the connecting rod from locking onto the fascia, which could lead to persistent and severe postoperative back pain. A dilating cannula was then inserted through the incision with the robotic arm until it reached the bone surface, and then the cannula core was withdrawn. The self‐tapping screw was then placed into the pedicle using a motorized drill under navigation, and the drill was withdrawn by reversing the gear. The next screw was selected at the workstation, the robotic arm was then moved to the next screw position, and the procedure was repeated until all the screws were placed.

##### Navigation Aid OLIF

**FIGURE 2:**
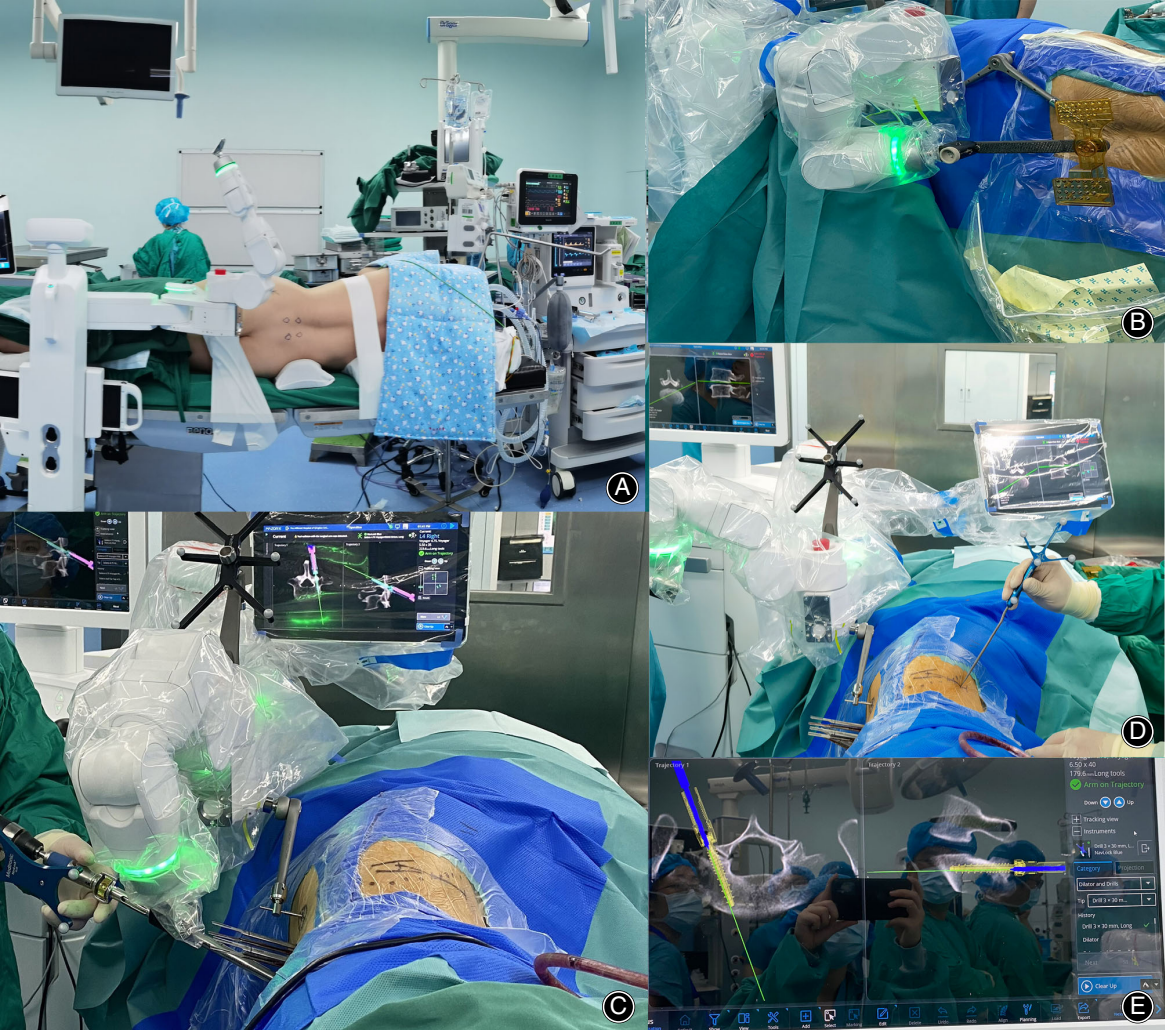
Main surgical procedures for the single‐position group. (A) Body positioning. The patient is placed in the right supine position, and the robot is connected to the end of the operating table. (B) Perspective matching after robot installation. It usually needs two matches of positive and oblique positions. (C) Robot‐guided screw placement without guidewire. (D) Navigation‐guided OLIF surgery. The probe provided by the system was used to locate and measure the intervertebral space. (E) Real time content for navigation on the display.

**FIGURE 3:**
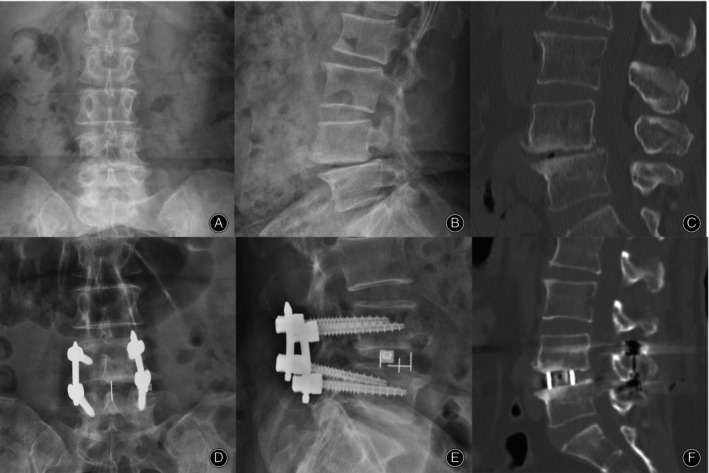
Comparison of a 65‐year‐old patient's preoperative and postoperative X‐ray and CT scan images in the single‐position group. Preoperative X‐ray (A, B) and CT scan images (C) showed that the patient suffered from lumbar spondylolisthesis with endplate degeneration; postoperative X‐ray (C,D) and CT scan images showed that the patient had good repositioning of the spondylolisthesis and no endplate damage.

After posterior screw placement, oblique anterior decompression and fusion procedures were performed. The probe of the robotic navigation system helped the surgeon make an appropriately positioned surgical incision and determine the segment when the lateral anterior aspect of the vertebral body was reached through the retroperitoneum and lumbar major muscle space. The probe can still be used to help verify the extent of decompression of the intervertebral disc. The posterior aspect of the cage was placed, and then the screw bar was locked (Figures 2 and 3).

#### Dual Position Group

##### Preoperative Planning

As in the single‐position group, we used preoperative computed tomography (CT) scans (scan layer thickness <1 mm, DICOM format) to obtain patient data and then transmitted them to the software of the robotic system for design. The surgical plan was transmitted to the workstation by the operating technician before the start of the procedure.

##### Positioning

Patients in the bipedal position group were first positioned in the classic lateral recumbent position, in the same position and fixation as in the unilateral group, and the iliac crest and the center of the disc at the target segment were marked on the skin under fluoroscopic guidance and then used to make the surgical incision. Subsequently, the oblique anterior disc was managed, and the cage was placed. After insertion, the patient was placed in the conventional prone position on the radiographic table with a sponge pad at the level of the chest and iliac crest, and the abdomen was suspended to reduce abdominal pressure. The patient was kept in a slightly flexed hip and knee position to reduce low back muscle tension. The number of lumbar segments and approximate arch locations were marked on the skin according to the body surface markings, and additional spinous markings were required for 2–3 segments above the operated segmental area as well as the bilateral iliac crest area.

##### Robot Installation, Registration

The iliac and sphenoid nails that were fixed in the marked area were used to check the stability of the fixation nails, after which the bridge was mounted to the three fixation nails and locked in place. The mark plate was positioned according to the system instructions, and fluoroscopy was performed with the patient in the orthogonal and oblique positions. The fluoroscopic images were transmitted to the robot workstation for matching, and the robot was then installed in the corresponding position on the bridge, which was generated by the robot system.

##### Screw Placement with Guide Wire

**FIGURE 4:**
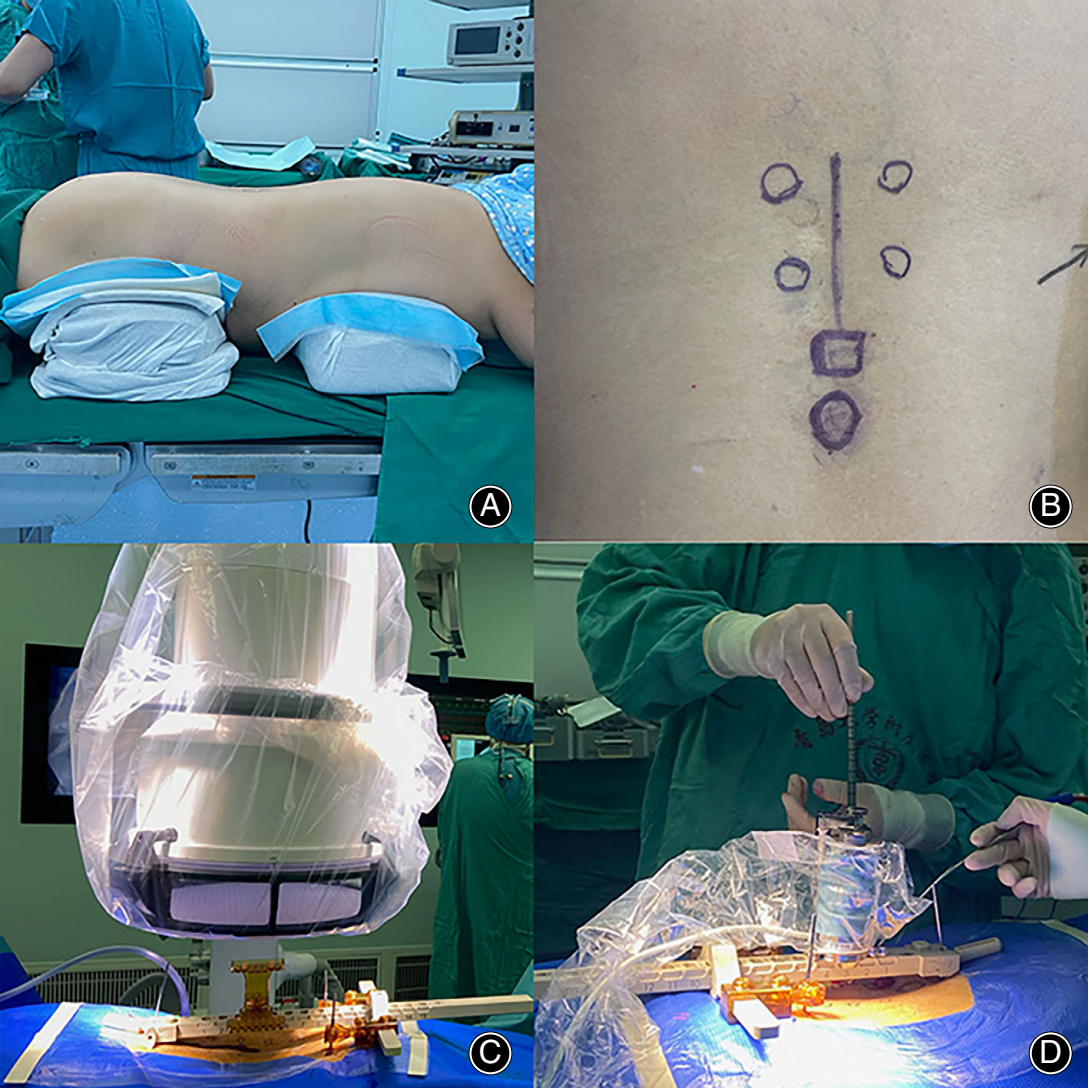
Main surgical procedure for screw placement in the dual‐position group. (A) Body positioning after lateral oblique lumbar interbody fusion. (B) Preoperative markings. (C) Perspective matching after robot installation. (D) Robot‐guided screw placement with guidewires.

**FIGURE 5:**
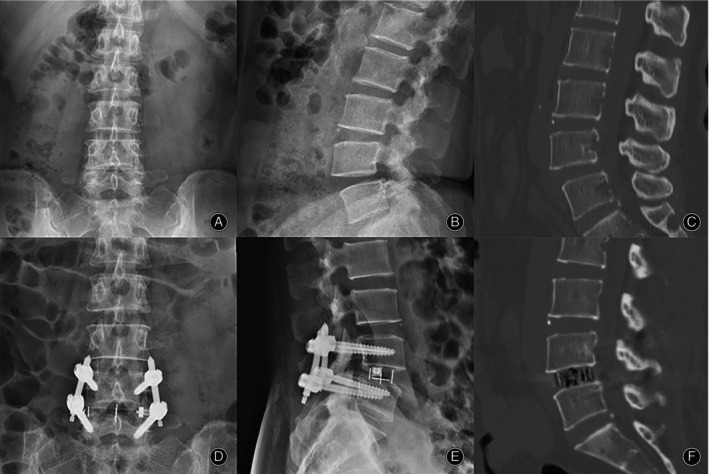
Comparison of a 57‐year‐old patient's preoperative and postoperative X‐ray and CT scan images in the bipedal position group.

After matching was completed, robot‐assisted screw placement was performed. After the robot selected the screw to be placed, the robot assembly was twisted, and the corresponding No. 1 or No. 2 trocar arm was installed according to the system prompts after the appropriate position was reached, followed by puncture with the robot‐equipped No. 11 special scalpel, the same as in the single position group. Then, an incision measuring approximately 3–4 cm was made in the deep fascia, and the incision was further dilated using a dilator. Then, the work was performed using wiretapping, the guidewire catheter was placed, and the guidewire catheter and trocar arm were withdrawn after insertion of the guidewire along the guidewire trocar. The guidewire was fixed, and the remaining guidewire was placed. Afterward, the screw was placed along the guide wire path using the nailer with the hollow nail, during which the guide wire was moved regularly to verify the correct orientation of the screw and to avoid the guide wire getting stuck in the path of the screw. After completion, the rod holder was used to thread the tail of the screw from the caudal end to the head end, deep into the deep fascia, and then the screw cap was locked (Figures 4 and 5).

### Accuracy Assessment

Postoperative 3D CT was routinely performed on each patient, with two surgeons trained and involved in the procedure taking measurements, recording data, according to the Gertzbein–Robbins classification.^12^ The accuracy of each screw was recorded. Briefly, the degree of deviation was classified as Grade A (completely within the pedicle), Grade B (notch ≤2 mm), Grade C (2 mm < notch ≤4 mm), and Grade D (notch >4 mm), depending on the distance from the pedicle border. Clinically acceptable slight deviations and notches of <2 mm make both grade A and grade B unnecessary for secondary intraoperative adjustment.

### Single‐Position Robot‐Assisted Versus Bipedal‐Position Robot‐Assisted OLIF Surgery

We collected data from 15 patients (*n* = 15) who underwent OLIF surgery with robotic assistance in a single position and 30 patients (*n* = 30) who also underwent OLIF surgery with robotic assistance in two different positions. Demographic data (sex, age, and BMI), clinical information (operative time, EBL, number of screws per case, number of fluoroscopies, and complications), and clinical outcomes (assessment of screw accuracy by postoperative CT scan, VAS scores for back pain and leg pain, and ODI scores) were compared between the single and bipedal position groups.

### Statistical Analysis

Statistical analyses were performed using SPSS 25.0 software for Windows (IBM Co, Armonk, NY, USA). Descriptive statistics are reported as the mean ± standard deviation, frequency or percentage when appropriate. Continuous data with a normal distribution were analyzed by Student's t test, and the Mann–Whitney U test was used for continuous data with a nonnormal distribution. The chi‐square test or Fisher's exact test was used for categorical variables. *p* < 0.05 was considered statistically significant.

## Results

### Demographic Characteristics

The mean age of the 15 (30%) patients in the unilateral group was 59.00 ± 12.72 years, including seven males and eight females. The mean age of the 35 (70%) patients in the double position group was 59.03 ± 13.32 years, including 11 males and 24 females. There were no significant differences in the demographic data between the two groups of patients (*p* > 0.05). More detailed information is presented in Table 1.

**TABLE 1:** Demographic data.

Indexes	Single position group (*n* = 15)	Bipedal position group (*n* = 35)	Statistical value	*p* value
No. of cases	15	35		
Sex				
Male	7	11	1.058	0.304^a^
Female	8	24		
Age (years)	59.00 ± 12.72	59.03 ± 13.32	0.060	0.953^b^
BMI (kg/m^2^)	26.31 ± 3.19	26.25 ± 2.94	0.064	0.949^b^
Spondylolisthesis severity				
Grade I	9	22	0.36	1.000^a^
Grade II	6	13		
Spondylolisthesis type				
Degenerative	12	26	0.005	0.942^a^
Isthmic	3	9		
Operated level				
L3‐4	1	3	0.000	1.000^a^
L4‐5	14	32		

*Note*: Data are presented as the mean ± standard deviation or frequency.

^a^ chi‐squared test

^b^ *t* test.

### Perioperative Parameters

As shown in Table 2, in our study, the mean operative time was significantly lower (*p* < 0.05) in the unilateral group (186.13 ± 45.62 minutes) than in the dual‐position group (389.14 ± 67.27 minutes), and the number of fluoroscopies was lower (6.2 ± 0.86) in the unilateral group than in the dual‐position group (7.74 ± 1.27) (*p* < 0.05). The estimated intraoperative blood loss was greater in the unilateral group (43.33 ± 18.38 mL) than in the dual‐position group (36.29 ± 14.16 mL), but there was no statistically significant difference between the two groups (*p* = 0.135).

**TABLE 2:** Perioperative parameters.

	Single position group (*n* = 15)	Bipedal position group (*n* = 35)	Statistical value	*p* value
EBL (mL)	43.33 ± 18.38	36.29 ± 14.16	1.493	0.135^a^
Operative time (min)	186.13 ± 45.62	389.14 ± 67.27	−12.399	<0.001^b^
Perspective Times	6.2 ± 0.86	7.74 ± 1.27	−4.9963	<0.001^b^

*Note*: Data are presented as the mean ± standard deviation.

EBL: estimated blood loss.

^a^ Mann–Whitney *U* test

^b^ *t* test.

### Clinical Information

A total of 200 screws were placed in both groups, and the surgical segments included L3 to L5. A total of 186 screws (93%) reached the grade A level, 14 screws (7%) reached the grade B level, and no screws reached grade C or D. Details are listed in Table 3. The percentage of screws reaching the grade A level was slightly higher in the single‐position group (96.67%) than in the bipedal‐position group (91.43%), but there was no significant difference between the two groups (*p* = 0.312). No serious complications, such as postoperative infection or dural tears, occurred in either group in this study. Complications mainly included myofascial pain and delayed wound healing, and no screw‐related complications occurred in either group.

**TABLE 3:** Screw distribution and accuracy information.

Encroachment grade	L3	L4	L5	Total	Accuracy (%)
A (in pedicle)	5	94	90	186	93
B (0 <breach ≤2 mm)	3	9	2	14	7
C (2 mm < breach ≤4 mm)	‐	‐	‐	‐	‐
D (breach >4 mm)	‐	‐	‐	‐	‐

*Note*: Data are presented as frequency.

### Effect Analysis

Back pain VAS scores were significantly lower in both groups, but the single‐position group had better outcomes at the 1‐week postoperative follow‐up (*p* < 0.05). Symptoms continued to be alleviated in both groups for more than 3 months after surgery, but there was no significant difference between the two groups. Leg pain VAS scores and ODI scores were similar in both groups and continued to improve during the follow‐up period, from 1 week postoperatively until 1 year postoperatively, with no significant difference between the two groups (*p* > 0.05). Further results are shown in Table 4 and Figure 5.

**Table 4:** Preoperative and Follow‐Up VAS and ODI Scores.

	Single position group (*n* = 15)	Bipedal position group (*n* = 35)	Statistical value	*p* value
VAS scores (back pain)				
Preoperation	6.93 ± 0.70	7.06 ± 0.80	0.457	0.648^a^
1 week	3.67 ± 0.90	4.34 ± 1.03	0.721	0.034^a^
3 months	2.20 ± 1.52	2.00 ± 1.33	0.467	0.485^b^
12 months	1.80 ± 1.27	1.66 ± 0.94	0.444	0.771^b^
VAS scores (leg pain)				
Preoperation	6.13 ± 1.73	6.29 ± 1.53	0.197	0.844^b^
1 week	3.80 ± 1.57	3.46 ± 1.69	0.679	0.497^b^
3 months	2.07 ± 1.10	2.00 ± 1.24	0.388	0.698^b^
12 months	1.33 ± 0.62	1.34 ± 0.54	0.024	0.981^b^
ODI				
Preoperation	30.73 ± 5.11	30.51 ± 5.65	0.129	0.898^b^
1 week	16.4 ± 2.35	16.54 ± 2.98	−0.164	0.870^b^
3 months	8.33 ± 1.80	8.11 ± 2.17	0.730	0.466^b^
12 months	5.33 ± 1.80	6.09 ± 1.579	1.114	0.265^b^

*Note*: Data are presented as the mean ± standard deviation.

^a^ Mann–Whitney *U* test

^b^ *t* test.

## Discussion

As far as we know, this study is the first report in China to use Mazor X robot to complete OLIF combined with pedicle screw implantation in lateral position. However, the difference between the lateral position and the traditional bipartite position is not clear. In our study, Mazor X robot‐assisted lateral position OLIF surgery resulted in a qualitative change in the surgery itself. We have achieved higher screw accuracy, a better intraoperative experience, and the same postoperative efficacy as double position surgery, while significantly reducing surgical time and fluoroscopy frequency.

### Perioperative Differences

In our robot‐assisted cases, we found a significant reduction in operative time due to no need for turning, repositioning, fluoroscopic positioning, or disinfection, which meant a shorter anesthesia time and less intraoperative patient movement, thereby reducing the risk of surgery. In addition, using the same position for fluoroscopy allowed simultaneous marking of the incision for the OLIF procedure and positioning of the segment required for screw placement. This step is expected to reduce the number of fluoroscopies per procedure. The results of the study showed that the robot‐assisted single‐position group had fewer fluoroscopies than the dual‐position group. Interestingly, postoperative assessment of screw accuracy showed that the robot‐assisted unilateral group had a significantly higher percentage of grade A placement (screws completely within the pedicle) in the robot‐assisted bimanual group, possibly because the screw placement procedure in the unilateral group was performed prior to the decompression and fusion step, which may have compensated for the discrepancy between the spinal structure of the patient's operative segment and the preoperative imaging data for OLIF.

### Screw Accuracy

In terms of screw accuracy to a passing (A + B) level, there was no significant difference between the single‐position group and the double‐position group (*p* > 0.05), indicating that changes in position do not affect accuracy. When performing lateral screw placement, what impressed us was that all our operations could be displayed on the screen of the robot in real time. We can simultaneously observe the position and angle of the screw on the lateral and cross‐section and show the difference from the preoperative plan. This allowed us to monitor and adjust the trajectory during screw placement. In addition, the robot supports single vertebral body matching, which means that in the OLIF operation, the operation of oblique anterior decompression will not affect the accuracy of screw placement. It has been suggested that the accuracy of robotic screw placement is only influenced by matching accuracy and its own accuracy.^17^, ^18^ However, current studies differ in terms of whether changes in position have an effect on screw accuracy, and in an analysis of the accuracy of 453 screws in 78 patients who received lateral placement in the prone position by Okuda, Ryuichiro et al. showed no statistically significant conceptual difference from the prone group.^19^ A meta‐analysis by Keorochana et al. showed no statistically significant difference between the lateral and prone positions in terms of intraoperative blood loss, radiotherapy dose, lumbar lordosis, incidence of complications, or need for reoperation,^20^ while a study by Fayed et al. showed that lateral position nailing had a higher error rate.^21^ In addition, most studies used only postoperative CT as a criterion for the presence and extent of bias. In clinical practice, for intraoperative misaligned K‐wires, the surgeon may choose to realign or change to freehand placement. Therefore, the actual rate of deviation may be higher than that derived from postoperative CT findings alone. In addition to TPS screws, CBT screws are included in the types of screw placement, and for this patient, we performed a TPS screw + CBT screw dual fixation scheme, which allows for easy design of a dual screw tract scheme in preoperative planning, reflecting the advantages of this system in preoperative planning. Compared with electromagnetic navigation assist systems, robotic assist has better stability, matching accuracy, and interference resistance.^22^

### Other Advantages

In addition to instrumentation, the robot‐assisted system has demonstrated impressive reliability in other situations where precise positioning is needed, such as when designing an OLIF incision, where conventional C‐arm fluoroscopy can only present a lateral two‐dimensional marker, while with the probe of the robotic navigation system, it is easier to locate the center of the disc and helps the surgeon correct during decompression and the placement of the fusion device. The robot navigation system makes it easier to locate the center of the disc and helps the surgeon correct the operating angle during decompression and fusion placement to achieve a three‐dimensional mark. In terms of postoperative complications, according to reports, postoperative complications of OLIF include abdominal organ injury, infection, lumbar sympathetic trunk injury, ureteral injury, etc.^23^, ^24^ In our cases, there was only one case of postoperative myofascial pain in the unilateral group, but the symptoms improved significantly after conservative treatment, which was consistent with the common postoperative complications of traditional percutaneous screw placement, but the positive rate was too low to be statistically significant.

### Clinical Efficiency

Regarding the postoperative outcome, patients in the single‐position group had significantly better VAS scores for back pain in the early period (1 week postoperatively) than those in the bipedal‐position group. We speculate that this may be caused by the reduction in intraoperative changes in patient position. The change in position in patients under general anesthesia who have just undergone OLIF stand‐alone surgery may lead to a currently unspecified hidden injury.

### Limitations and Prospect of Clinical Application

This study has several limitations. Since this technique is new in China, there are only a few cases, which may cause bias in statistical analysis. Therefore, a larger sample size and multicenter study are needed to further verify the results of this study. However, the unique full body position support and excellent navigation system of this robot have immeasurable advantages in reducing the learning curve, improving the efficiency and safety of surgery.

## Conclusion

In this study, robot‐assisted OLIF with lateral posterior screw placement in the lateral position had high accuracy and a low complication rate. Robot‐assisted lateral screw placement has the same accuracy as prone positioning. Mono‐position surgery resulted in significantly shorter operative times as well as fewer fluoroscopies. In addition, the early postoperative outcome was slightly better in the single position group than in the bipedal position group, and the long‐term outcome was not significantly different from that of the bipedal position group. We are optimistic about the future application of this robot‐assisted technique.

## Conflict of Interest Statement

The authors declare that the research was conducted in the absence of any commercial or financial relationships that could be construed as a potential conflict of interest.

## Author Contributions

Wenhao Zhao conceived and designed the study. Hao Zhang and Antao Lin collected the data. Yan Wang and Jialuo Han performed the statistical analyses. Jianwei Guo and Wenhao Zhao prepared all figures and tables. Wenhao Zhao and Chuanli Zhou wrote the manuscript. Xue‐xiao Ma was the supervisor of the project, revised the manuscript, and was responsible for the study. All authors reviewed and revised the final manuscript.

## Ethics Statement

Our study was approved by the Ethics Committee of Affiliated Hospital of Qingdao University (Approval: QYFY WZLL 28119). All methods were carried out in accordance with relevant guidelines and regulations.

## Data Availability

The data that support the findings of this study are available from the corresponding author upon reasonable request.
